# Position-controlled quantum emitters with reproducible emission wavelength in hexagonal boron nitride

**DOI:** 10.1038/s41467-021-24019-6

**Published:** 2021-06-18

**Authors:** Clarisse Fournier, Alexandre Plaud, Sébastien Roux, Aurélie Pierret, Michael Rosticher, Kenji Watanabe, Takashi Taniguchi, Stéphanie Buil, Xavier Quélin, Julien Barjon, Jean-Pierre Hermier, Aymeric Delteil

**Affiliations:** 1 Université Paris-Saclay, UVSQ, CNRS, GEMaC, Versailles, France; 2 Laboratoire de Physique de l’École Normale Supérieure, ENS, Université PSL, CNRS, Sorbonne Université, Université de Paris, Paris, France; 3 Research Center for Functional Materials, National Institute for Materials Science, Tsukuba, Japan; 4 International Center for Materials Nanoarchitectonics, National Institute for Materials Science, Tsukuba, Japan

**Keywords:** Two-dimensional materials, Single photons and quantum effects

## Abstract

Single photon emitters (SPEs) in low-dimensional layered materials have recently gained a large interest owing to the auspicious perspectives of integration and extreme miniaturization offered by this class of materials. However, accurate control of both the spatial location and the emission wavelength of the quantum emitters is essentially lacking to date, thus hindering further technological steps towards scalable quantum photonic devices. Here, we evidence SPEs in high purity synthetic hexagonal boron nitride (hBN) that can be activated by an electron beam at chosen locations. SPE ensembles are generated with a spatial accuracy better than the cubed emission wavelength, thus opening the way to integration in optical microstructures. Stable and bright single photon emission is subsequently observed in the visible range up to room temperature upon non-resonant laser excitation. Moreover, the low-temperature emission wavelength is reproducible, with an ensemble distribution of width 3 meV, a statistical dispersion that is more than one order of magnitude lower than the narrowest wavelength spreads obtained in epitaxial hBN samples. Our findings constitute an essential step towards the realization of top-down integrated devices based on identical quantum emitters in 2D materials.

## Abstract (web summary)

Accurate control of the spatial location and the emission wavelength of single photon emitters (SPEs) in van der Waals materials is a crucial yet challenging endeavour. Here, the authors use an electron beam to generate SPE ensembles in high purity synthetic hBN with enhanced spatial accuracy and emission reproducibility.

## Introduction

The technological control of van der Waals materials is continually expanding, motivated by the possibility of realizing increasingly complex hetero- and nanostructures of minimal thickness. The considerable variety of impacted fields of physics^1,2^ has been including solid-state quantum optics^3^ since the discovery of single photon emission in WSe_2_^4–8^ and hBN^9^. In the latter material, quantum emission is associated with point defects that were long thought to be of the intrinsic kind, although carbon impurities have been shown to play a role in the structure of at least part of the observed SPEs^10^. Their emission is found to be bright, stable^11,12^ and spectrally narrow^13,14^, and persists up to room temperature and above^15^. They however suffer from large discrepancies between their emission wavelengths, which are typically found between 550 and 850 nm^16,17^. Epitaxial hBN grown by chemical vapour epitaxy has been recently shown to lead to a narrowing of the spectral distribution down to about 20 nm (75 meV) around a centre wavelength of 585 nm^10,18^. Moreover, the SPEs appear in most cases at random locations in the crystal, although often preferentially close to the flake edges^19^. Effort towards controlling their position has included the use of focused ion beam^20^, as well as strain through exfoliation on patterned substrates^21^, but the emitters obtained with these methods exhibit large variations in their number, emission wavelength and optical properties. Moreover, the latter method results in limited possibilities of subsequent integration. In the 2D material MoS_2_, deterministic positioning with high precision ( ~ 10 nm) has been achieved^22,23^ using He ion beam, but at the current stage the generated SPEs suffer from low count rates and large linewidths, which constitutes a major drawback for applications to photonic quantum information.

Here, we demonstrate the activation of colour centres at chosen locations using the electron beam of a commercial scanning electron microscope (SEM). Electron irradiation has already been shown to increase the formation probability of the SPEs^16,19,24^, but to date has never been the basis of a process that allows to activate SPEs at preselected locations in hBN. We show that our local irradiation process activates SPE ensembles with a submicrometric precision. The SPEs exhibit a strongly reduced ensemble linewidth with respect to prior work on 2D materials. We investigate individual quantum emitters and demonstrate advantageous photophysical properties, with in particular a high stability of both fluorescence intensity and centre wavelength fluctuations. Our work paves the way to top-down fabrication of integrated devices based on SPEs in hBN.

## Results

### Generation and characterization of SPE ensembles

**Fig. 1:**
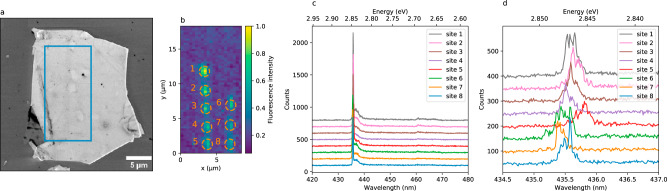
Activation of localized ensembles of SPEs on a hBN flake. **a** SEM image of a high-purity hBN flake of about 15 × 20 *μ*m and 60 nm thickness. **b** Confocal map of the irradiated zone (blue rectangle in  **a**) with eight irradiation spots (orange dashed lines). (**c**) and (**d**) Low-temperature spectra of the eight spots with two different spectral resolutions, showing a reproducible ZPL within 0.7 nm.

We use high purity hBN synthesized at high pressure, high temperature (HPHT)^25^, of which we exfoliate single flakes of a few tens of nanometres thickness on a silicon substrate, either with or without a top 285 nm SiO_2_ layer. The flakes are irradiated using an electron beam of 15 keV acceleration voltage, under a current of 10 nA. We first focus on the sample with the SiO_2_ epilayer, which we refer to as sample 1. For this sample, the beam is adjusted to be about 33 nm diameter, to compromise between maximizing both the interaction cross-section and the localization accuracy. The irradiation time is fixed at 1000 s per irradiated spot. No additional treatment is performed on the sample. After the irradiation process, the sample is subsequently characterized in photoluminescence (PL) in a confocal microscope, either at room temperature or at cryogenic temperature down to 5 K. The SPEs are non-resonantly excited using a laser at 405 nm, in pulsed or continuous wave regime. Figure 1a shows a SEM image of one of the irradiated flakes (of thickness 60 nm), together with a low temperature (5 K) confocal fluorescence map of the irradiated zone (Fig. 1b). Emission from ensembles of colour centres is observed in all irradiated spots, within a radius close to that of the electron beam (see Supplementary note 1) and thus showing that the emitters are localized in a volume of about 3.5 × 10^−2^ μm^3^ ≈ 0.4*λ*^3^, where *λ* ≈ 435 nm is the emission wavelength. The low-temperature spectra associated with the irradiated sites are shown Fig. 1c, d with two different resolutions. On the coarse resolution spectra (Fig. 1c; see also Supplementary note 2), the overall common spectral shape of the SPEs can be observed: they exhibit a sharp zero-phonon line (ZPL) around 2.846 eV (435.7 nm) that concentrates about 40% of the light emission, as well as an adjacent acoustic phonon sideband (45%) and two phonon replica, respectively red-shifted by 155 and 185 meV (15%). The high resolution spectra, centred around the ZPL, is shown Fig. 1d, where ensembles of discrete lines are observed. Keeping the above-mentioned irradiation parameters, we have overall realized 26 irradiation spots on 3 flakes. All of them gave rise to small ensembles of similar emission wavelength. The ensemble distribution, inferred from the PL spectra of all 26 spots, has a full width at half maximum (FWHM) of 3 meV (see Supplementary note 3), which is an order of magnitude narrower than the state of the art in 2D materials^23^. We estimate the number of emitters per site to be of order of a few tens, as confirmed by photon correlation measurements (see Supplementary note 4). Remarkably, no colour centre, neither at 435 nm nor in the more usual wavelength range 550–850 nm, has been observed elsewhere on the flakes, although broad emission can be measured near the edges or close to flake defects. Interestingly, we note that light emission around 435 nm has already been observed in hBN as reported by Shevitski et al.^26^. In the latter work, however, blue emission could solely be observed in cathodoluminescence and did neither respond to laser excitation, nor exhibit any antibunching behaviour in the photon statistics. Nonetheless, it is likely that the SPEs we report here are of the same nature—we presume that, in our case, we are able to activate the response of the emitters to photoluminescence owing to our electron irradiation parameters being very different from those used in^26^, where the electron irradiation dose is several orders of magnitude smaller. The necessity of a relatively high dose is compatible with the scenario of a dissociation of a pre-existing defect induced by the electron beam, followed by a sufficient migration of the produced species to lead to a stable optically active defect. We also mention that our irradiation procedure did not lead to SPE activation in other sources of hBN grown at atmospheric pressure (see Methods), consistently with Shevitski et al.^26^, suggesting a physical origin of the SPEs related either to the HPHT growth conditions or to the specific solvent precursor used during the hBN synthesis.

### Individual SPE photophysics

**Fig. 2:**
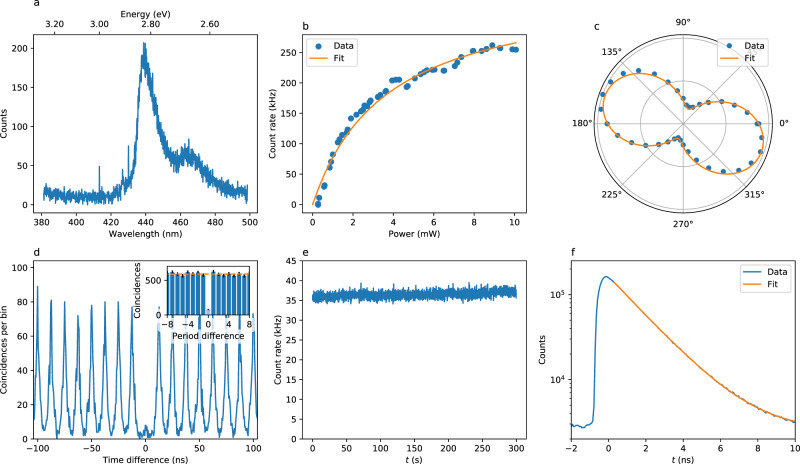
Photophysical characterization of an individual SPE at room temperature. **a** Emission spectrum of SPE_1_, showing a main peak centred at 440 nm (ZPL) and a phonon replica at 465 nm. **b** Count rate as a function of the laser power in cw regime. The orange curve is a fit to the data, from which we extract a saturation power of 3.7 mW and a maximum photon detection rate of 3.6 × 10^5^  Hz. **c** Count rate as a function of the angle of a polarizer placed before the detector, showing linearly polarized emission. The orange curve is a sine fit of the data. **d** Photon correlations in pulsed regime measured with 315 μW excitation power and 80 MHz repetition rate, yielding *g*^(2)^(0) = 0.12 ± 0.01 and thus demonstrating single photon emission. Inset: period-wise integrated coincidences (error bars: 1 standard deviation). The dashed orange line denotes the classical limit. **e** Time trace of the photon detection rate with 100  ms binning, calculated from the same raw data as (**d**). **f** Fluorescence decay in logarithmic scale, extracted from the same raw data as (**d**). The orange curve is an exponential fit to the data, yielding *τ* = 1.85 ns.

In order to investigate the individual properties of the colour centres, we have performed additional irradiations on sample 2, with a reduced exposition time (either 300 or 600 s) and a slightly larger electron beam (~1 μm diameter) on a thinner flake (~30 nm thickness). The irradiations yielded SPEs, some of which we could characterize individually (see Supplementary note 5). The emitted light is collected by an air objective of NA 0.95, and detected using avalanche photodiodes or a spectrometer (see Methods). Figure 2 shows the typical room temperature photophysical properties of a single representative colour centre, termed SPE_1_. The emission spectrum (Fig. 2a) shows that the emission mainly occurs in a ZPL centred at 440 nm, slightly red-shifted as compared with the low temperature emission. The linewidth of the ZPL is 12 nm. An optical phonon replica is visible around 465 nm. Figure 2b shows the count rate as a function of the laser power. The emission exhibits a saturation behaviour characteristic of two-level systems. We detect up to ~2.5 × 10^5^ photons per second when the SPE is excited above saturation. We fit the data with the standard power dependence of a two-level system fluorescence *I*(*P*) = *I*_sat_/(1 + *P*_sat_/*P*), yielding a saturation power of 3.7 mW and a saturation count rate of 0.36 MHz. This value is limited by the predominant emission of the SPE towards the high index absorptive silicon substrate and could be improved by a factor ~10 by collecting through a transparent substrate using an oil immersion objective, or by integrating the SPEs in a photonic structure. Figure 2c shows the emission polarization data of SPE_1_. The emission is linearly polarized, suggesting a single dipole transition linearly oriented in the basal plane of the hBN crystal. We have performed second-order correlation measurements in pulsed regime to establish the quantum character of light emitted by SPE_1_. Figure 2d shows the results we obtained, with a value of *g*^(2)^(0) = 0.12 ± 0.01 without background correction. This clear antibunching unequivocally demonstrates single photon emission from the colour centre. The count rate is stable over time, as can be observed on Fig. 2e, with no blinking or bleaching observed at timescales ≥1 ms. The absence of blinking at shorter timescales is ensured by second order correlations at intermediate timescales (see Supplementary note 7). Finally, Fig. 2f shows a fluorescence decay measurement, together with an exponential fit of the data. The lifetime of the excited state is found to be 1.85 ns, of the same order of magnitude as other families of SPEs in hBN.

### Statistical dispersion of individual SPE properties at room temperature

**Fig. 3:**
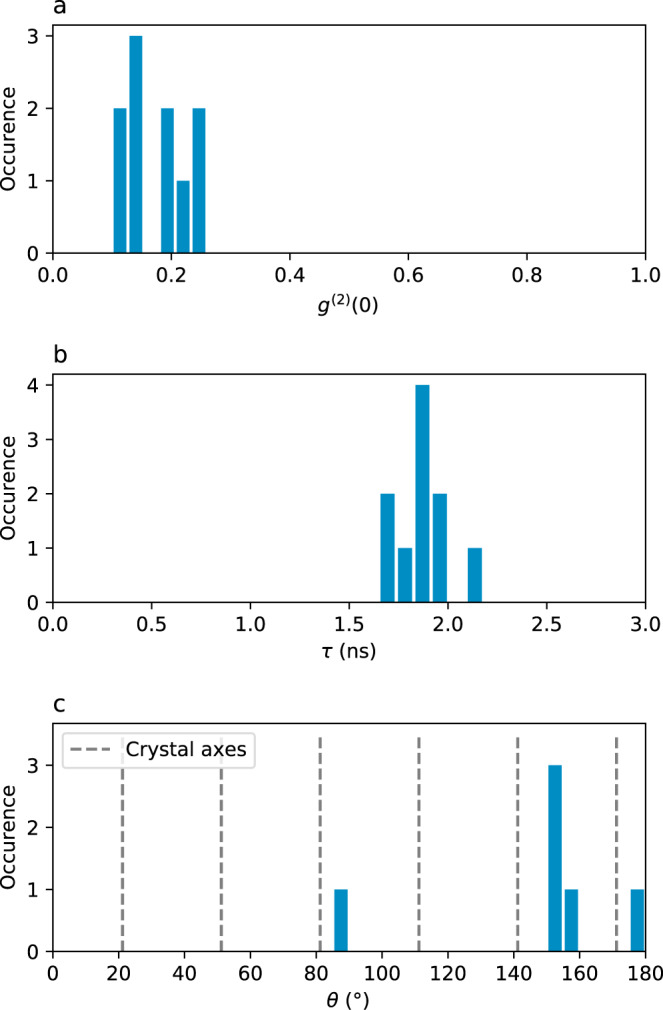
Statistical dispersion of individual SPE properties. **a** *g*^(2)^(0) of 10 SPEs, showing single-photon emission. **b** Fluorescence lifetime *τ* of the same 10 individual SPEs, with a mean value of 1.87 ns. **c** Polarization axis of the emission of 6 individual SPEs on the same flake, showing that most SPEs emit with a similar polarization direction.

We have performed similar measurements on 10 SPEs on two flakes (labelled SPE_1_ to SPE_10_). Figure 3 shows the statistical dispersion of the associated physical quantities. The value of *g*^(2)^(0) (without background correction) is found between 0.1 and 0.25, as shown Fig. 3a, mainly limited by fluorescence background and emission from nearby SPEs. Figure 3b shows the statistical spread of the fluorescence lifetime, which is centred around 1.87 ns with a standard deviation of 0.14 ns. Finally, the polarization angle of the emission from 6 SPEs on the same flake, to ensure a common crystalline orientation, is shown Fig. 3c. Although their directions seem correlated, they do not coincide with crystal axes. Additionally, we note that we did not observe any measurable variation of the ZPL wavelength at room temperature. These results show that the irradiation process yields SPEs with considerably homogeneous properties.

### Low-temperature spectroscopy of individual SPEs

**Fig. 4:**
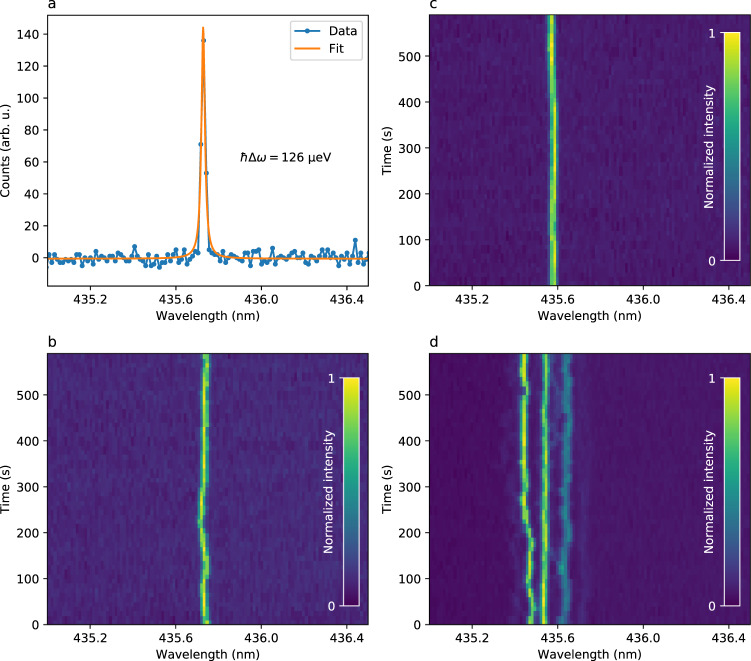
Spectral properties of individual SPEs at low temperature (5 K). **a** High-resolution spectrum of SPE_1_ ZPL, showing a resolution-limited line at 435.73 nm. **b** Spectral diffusion of SPE_1_ ZPL during 600 s. The standard deviation of the centre wavelength over time is found to be 45 *μ*eV, as determined by Lorentzian fits of the data. **c** Spectral diffusion of another SPE (SPE_2_) and **d** spectral diffusion of an ensemble of three SPEs, with uncorrelated fluctuations of different magnitudes. All SPEs are excited with 1 mW cw laser light at 405 nm.

The spectral properties of individual SPEs at low temperature have been further investigated, and are depicted Fig. 4. For most SPEs, the ZPL linewidth appears to be limited by our spectrometer resolution (~150 *μ*eV), which is the case for SPE_1_ as shown Fig. 4a. By measuring the emission spectrum as a function of time, we are able to observe the spectral diffusion of the ZPL. Figure 4b shows the result in the case of SPE_1_. We can observe fluctuations of the centre wavelength at timescales of a few seconds, with a standard deviation of 45 *μ*eV. The spectral diffusion of other SPEs is shown Fig. 4c and d. The standard deviation of the line positions over time typically lies in the range 10 to 50 *μ*eV (2.5 to 12 GHz), although some SPEs with larger fluctuations (a few 100 s of *μ*eV) have also been encountered. These values are in the very low range of values usually observed for SPEs in hBN under non-resonant excitation, and could be further improved using resonant excitation^14,27^. The spectral diffusion, attributed to charge fluctuations in the close environment of the defect, suggests that the emission is sensitive to static electric field, thus opening the way to dc-Stark tuning of the emission line using, for instance, graphene electrodes^28^. Given the natural spectral proximity of the emission from different SPEs, the possibility to electrically tune the emission wavelength could potentially allow to bring any pair of SPEs to resonance, enabling quantum interference of photons emitted by distinct SPEs.

## Discussion

In summary, we have demonstrated the possibility to activate SPEs in high-purity hBN at deterministic locations using the electron beam of a commercial SEM. This accessible process is well adapted to potential large-scale or industrial applications. The photophysical properties of the SPEs are advantageous and substantially replicable. In particular, the reproducibility of the emission line has no equivalent in 2D materials, and could open the way to quantum interference between distinct emitters. The relatively short emission wavelength opens the way to miniaturized on-chip applications, while still lying in the technology-friendly visible range. At low temperature, the spectral mismatch between the ZPL and the acoustic phonon sideband opens the way to the demonstration of indistinguishable photon emission by filtering out the incoherent contribution. However, while we have shown that antibunching persists up to room temperature, the emission becomes incoherent. Therefore, demonstration of room-temperature photon indistinguishability would imply to reach non-trivial cavity quantum electrodynamics regimes^29,30^ that would entail an accurate coupling of the SPEs to a microcavity. Our work brings fundamental questions on the precise nature of the colour centres and on the physical mechanism that renders them optically active upon electron irradiation, that will motivate both further experimental investigations and theoretical studies. On the technological side, it will be desirable to settle methods allowing to deterministically obtain a single SPE per irradiation spot. Such process could for instance make use of in-situ cathodoluminescence measurements^31^ during the irradiation process, heralding successful activation of a colour centre. This could in turn enable deterministic coupling of individual SPEs to photonic^32^ or plasmonic^33^ nanostructures. We expect our research to bring new possibilities to the field of quantum optics in 2D materials, that could yield applications in nanophotonics, integrated quantum optics, and quantum information science.

## Methods

### Sample fabrication

High-purity hBN was grown under high pressure/high temperature using barium boron nitride (Ba_3_B_2_N_4_) as a solvent system. The hBN flakes were obtained by mechanical exfoliation of bulk material on commercial silicon substrates. We used two different exfoliation methods for the two samples: for sample 1, the hBN has been exfoliated using two 3 mm thick polydimethylsiloxane (PDMS) stamps on a SiO_2_/Si substrate (with 285 nm SiO_2_ epilayer), and for sample 2 we used Scotch tape to exfoliate on a Si substrate. This allows to rule out the role of a specific residue in the SPE creation process. Prior to the exfoliation, the substrates were cleaned using acetone for 5 min, isopropyl alcohol for 5 min, followed by 5 min of 30 W oxygen plasma treatment. Two control samples grown at atmospheric pressure have also been used: a APHT (atmospheric pressure, high temperature) sample grown in KSU (Kansas, USA) using Ni/Cr solvent^34^, and a sample grown in LMI (Lyon, France) using PDC (polymer derived ceramics)^35^. Both samples have been exfoliated using adhesive tape on a SiO_2_/Si substrate. The SEM imaging and the electron irradiations were performed in a commercial SEM (JEOL 7001F). The flake thicknesses were measured with an atomic force microscope.

### Optical characterization

For room temperature characterization, the sample was placed in a confocal microscope with an air objective of NA 0.95. Low-temperature characterization was done in a closed-cycle cryostat and a low-T objective of NA 0.8 was used. In both cases, the sample was placed on three-axis piezo positioners. A 405 nm laser diode was used to excite the SPEs, either in continuous wave or in pulsed regime (pulse length ~200 ps, repetition rate 80 MHz). A dichroic mirror (cutoff wavelength 414 nm) and a fluorescence filter allowed to suppress back-reflected laser light. The signal was fibre-coupled to either a grating spectrometer (Princeton Instruments) or avalanche photodiodes (Micro Photon Devices) with 30% collection efficiency in the relevant wavelength range, and the detection event was recorded using a time-tagged single photon counting module (PicoQuant). In the photon correlations measurements, only the photons emitted after the laser pulse have been recorded in order to avoid double excitation events caused by the finite laser pulselength.

## Supplementary information

Supplementary Information

## Data Availability

The data generated in this study are available at 10.5281/zenodo.4768457.
