# Supplementary material for: Position-controlled quantum emitters with reproducible emission wavelength in hexagonal boron nitride
Source: Nat Commun. 2021 Jun 18;12:3779. doi: 10.1038/s41467-021-24019-6 (PMC8213715; doi:10.1038/s41467-021-24019-6)
Supplement: Supplementary file 1 — Supplementary Information [file 41467_2021_24019_MOESM1_ESM.pdf]

# Supplementary Information

# Position-controlled quantum emitters with reproducible emission wavelength in hexagonal boron nitride

Clarisse Fournier, Alexandre Plaud, Sébastien Roux, Aurélie Pierret, Michael Rosticher, Kenji Watanabe, Takashi Taniguchi, Stéphanie Buil, Xavier Quélin, Julien Barjon, Jean-Pierre Hermier and Aymeric Delteil

## Supplementary note 1: Characterization of the spot diameter

In the first round of irradiation (sample 1, corresponding to the figure 1 of the main text), the electron beam diameter has been calibrated by irradiating the substrate and imaging the modification it yielded to the secondary electron imaging signal in the SEM (supplementary figure 1a). The irradiation leads to a reduction of the signal from the substrate within a slightly elliptic Gaussian spot. We summed the spot image along two orthogonal direction and fitted the result to obtain a mean FWHM of 315 nm (supplementary figure 1b).

The spatial distribution of the PL signal from SPE ensembles has been characterized with a similar procedure: the room-temperature PL signal (supplementary figure 1c) was summed along two orthogonal direction and the resulting Gaussian shape was fitted, yielding the FWHM of the intensity distribution. Statistics from 10 irradiation spots lead to an average FWHM of 850 nm. This value is slightly greater than the spot size convolved by the emission wavelength (which yields 550 nm). The mismatch could be attributed to the relatively long duration of the irradiations leading to drifts in the beam position and therefore to an effective broadening of the spot, as well as to secondary electrons creating colour centres in the close vicinity of the spot.

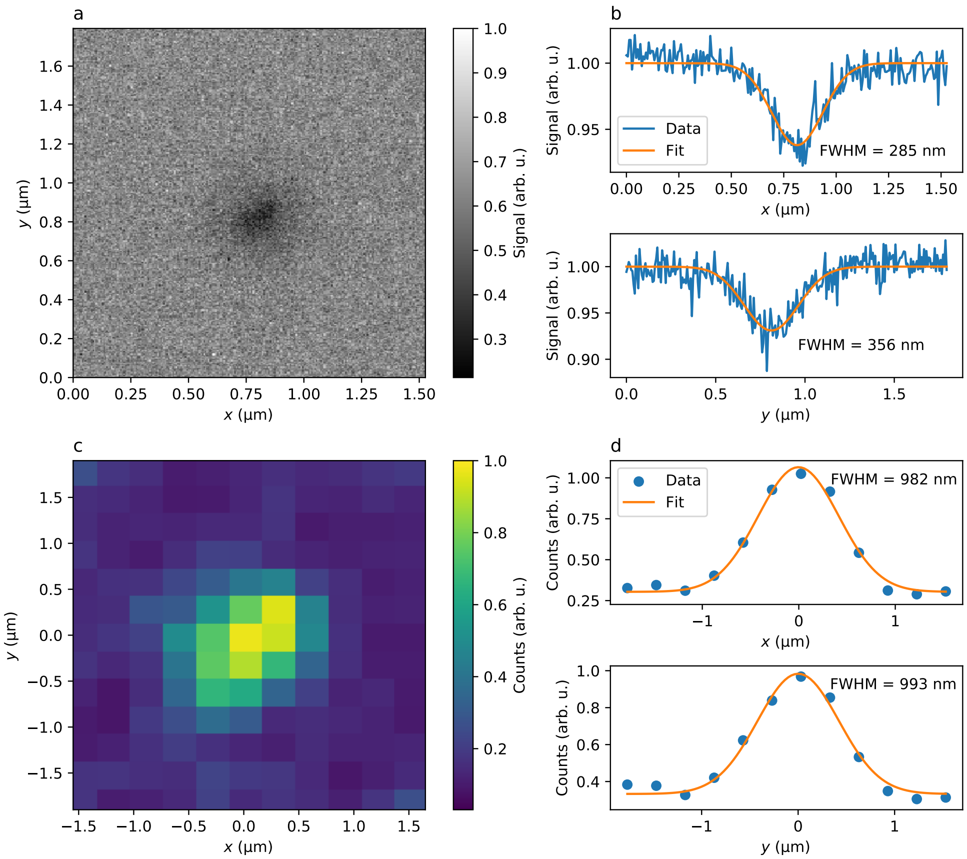


Supplementary figure 1: (a) SEM image of an irradiation on the $SiO_2$ substrate. (b) Sum along $x$ (upper panel) and $y$ (lower panel) from which we estimate the beam dimensions. The orange lines are Gaussian fits of the data. (c) Room temperature photoluminescence confocal map of a SPE ensemble from an irradiation spot. (d) Cuts along $x$ (upper pannel) and $y$ (lower pannel) from which we extract the characteristic dimensions of the SPE ensemble.

## Supplementary note 2: Spectral shape and phonon replica

In order to better appreciate the spectral lineshape of the SPEs, we show supplementary figure 2 an ensemble spectrum – calculated as the sum of the spectra shown supplementary figure 1c of the main text – in logarithmic scale. We can distinguish an acoustic phonon sideband [1], as well as two phonon replica, labelled LO1 and LO2, respectively redshifted by 155 meV and 185 meV relatively to the ZPL. We note that these values are slightly lower than usually observed for other SPEs [2], with however a similar splitting of about 30 meV between LO1 and LO2.

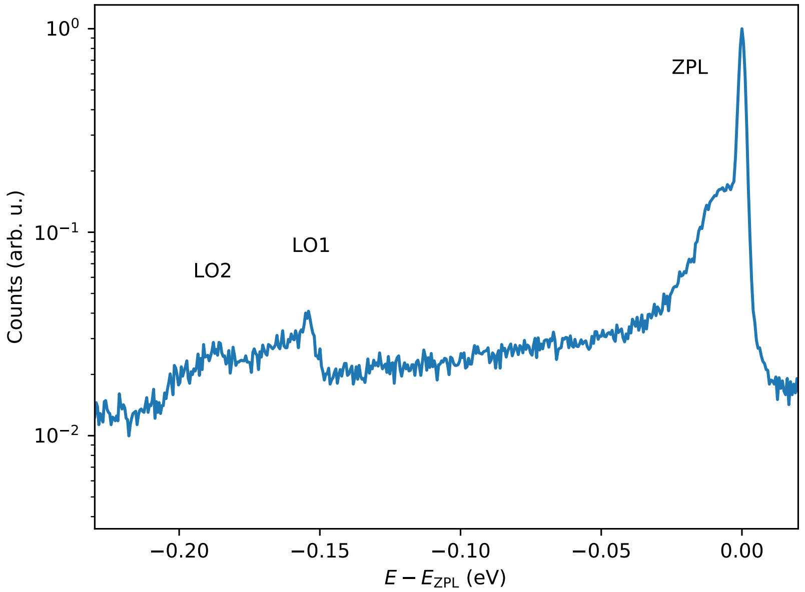


Supplementary figure 2: Ensemble spectrum plotted as a function of the relative energy from the ZPL maximum ($E_{\mathrm{ZPL}}$).

## Supplementary note 3: Ensemble linewidth

To estimate the probability distribution of the ZPL wavelength, we have summed the spectra taken over the 26 irradiations we performed on sample 1. This corresponds to several hundreds of emitters (see next section). The ensemble spectrum we obtain (supplementary figure 3) was fitted with a sum of two Gaussian functions, respectively associated with the ZPL and the acoustic phonon sideband. The fit parameters provide the ZPL centre wavelength (435.78 nm) and FWHM (0.46 nm, *i.e.* 3.0 meV). The phonon sideband has a centre wavelength of 437.0 nm and a FWHM of 2.9 nm, *i.e.* 19 meV.

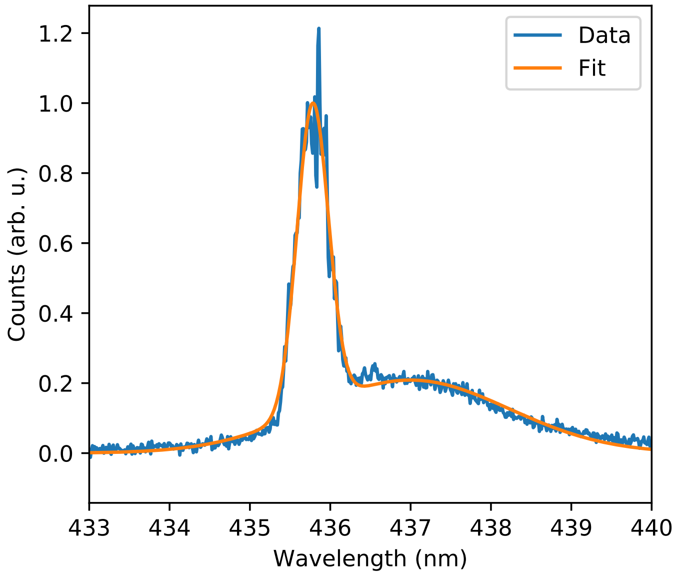


Supplementary figure 3: Ensemble spectrum summed over 26 irradiation sites on sample 1. The Gaussian fit provides a 3 meV FWHM for the ZPL distribution.

# Supplementary note 4: Estimation of SPE number in sample 1

The number of emitters in the irradiated spots can be estimated by combined low-temperature PL spectroscopy and second-order correlation of the ensemble emission. Fig 4a-d shows PL spectra of four typical ensembles at 5 K. Although counting the number of SPEs is not directly possible due to the too close proximity of the ZPLs, several discrete lines can be observed, suggesting that the number of individual lines is at least of a few units. Fig 4e-h shows the room-temperature second order correlation of the same ensembles, with $g^{(2)}(0)$ typically found around 0.95. As a reference, a $g^{(2)}(0) = 0.95 \pm 0.02$ corresponds to a number of $\sim 20$ ideal single-photon emitters – in general, the $g^{(2)}(0)$ of $N$ ideal single-photon sources is given by $1 - 1/N$. We note that the number estimated from $g^{(2)}(0)$ constitutes an upper bound of the SPE number, since background, dark counts and other nonidealities can only degrade the antibunching.

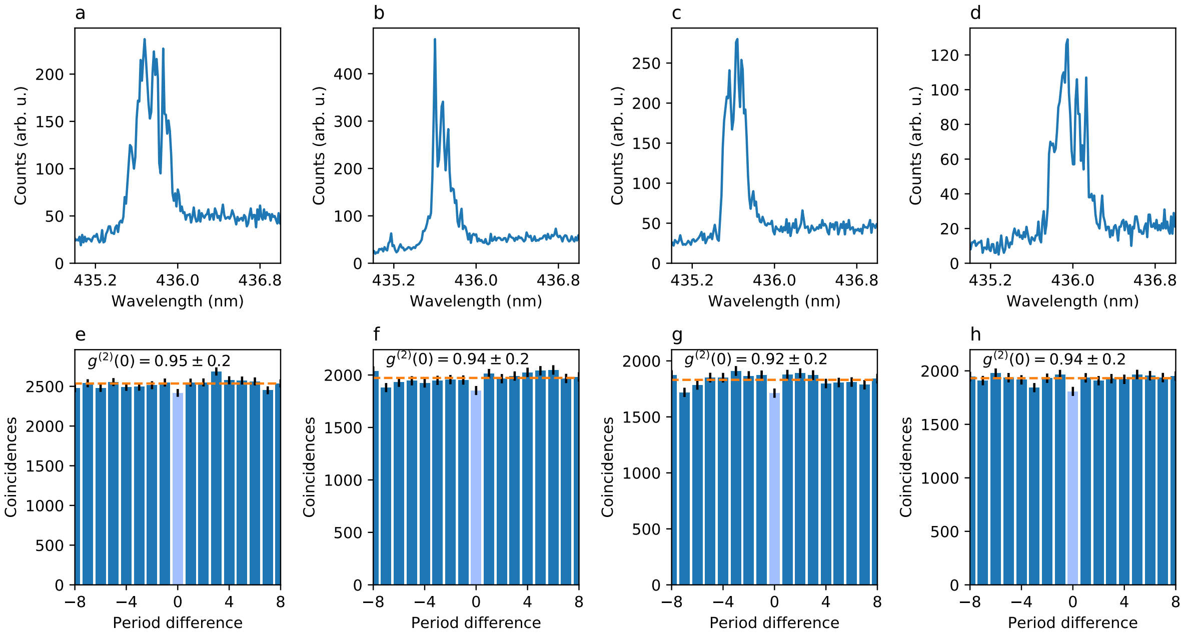


Supplementary figure 4: (a)-(d) Low-temperature PL spectra of four SPE ensembles and (e)-(f) corresponding RT second-order correlation demonstrating some degree of antibunching. The orange dashed line denotes the classical limits. The value of $g^{(2)}(0)$ is indicated above each histogram. The error bars represent 1 standard deviation as calculated from Poissonian statistics of the photon counting events.

.

## Supplementary note 5: Isolation of individual SPEs

As mentioned in the main text, a 30 nm flake has been irradiated with a lower dose, spread over larger areas. Supplementary figure 5 shows a confocal map of two irradiated spots with 10 min irradiation time. The orange circles denote the nominal beam diameter (FWHM $\sim 1\ \mu$m) used for this flake. With these irradiation parameters, individual SPEs can be isolated at the vicinity of the spots as indicated by the red circles. The rest of the luminescence originates from small ensembles of two SPEs or more, as confirmed by both $g^{(2)}$ measurements and spectroscopy, with the presence of several ZPLs.

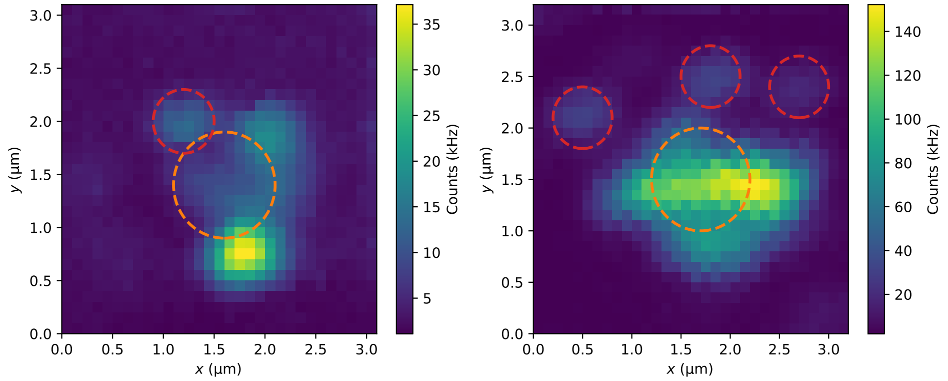


Supplementary figure 5: Room-temperature confocal maps of two irradiations. The orange circle is the estimated position of the electron beam and the red circles point individual SPEs.

## Supplementary note 6: Flake characterization

On supplementary figure 6 we show optical microscopy, scanning electron microscopy (SEM) and atomic force microscopy (AFM) of an irradiated flake from sample 1, with 10 irradiated spots (with irradiation parameters as described in the main text). The effect of the electron irradiation is not measurable in optical microscopy. On the AFM map, the only trace of the irradiation process on the 60 nm thick flake is a slightly thinner ($\sim$ 2–3 nm) zone within a 2.5 $\mu$m radius around the irradiations – which we also observe if we directly irradiate the substrate. The SEM image, in turn, reveals a brighter contrast a couple of micrometres around the irradiation spots. The confocal PL scan reveals ten bright spots at the position of the irradiated sites.

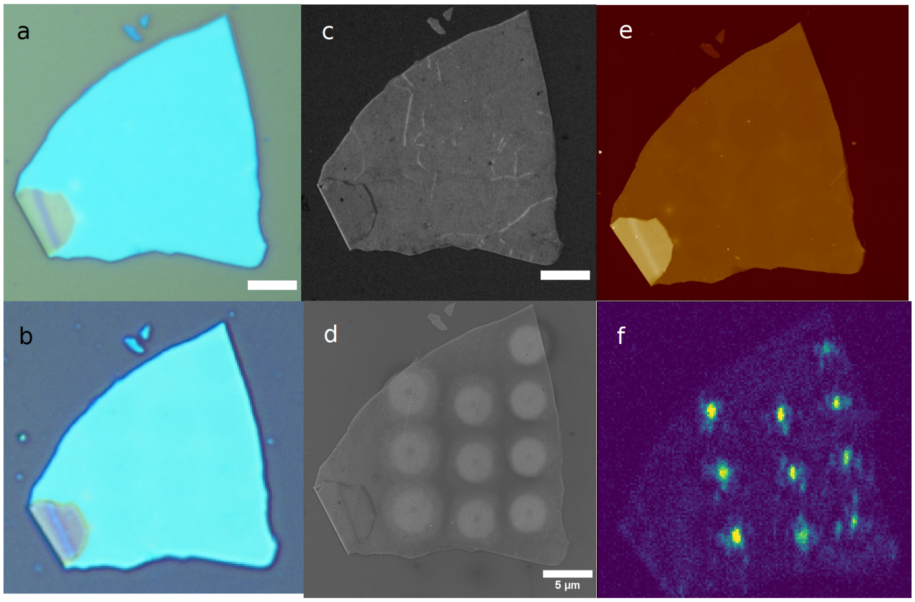


Supplementary figure 6: (a) Optical microscope image of the flake before irradiation, and (b) after irradiation. (c) Scanning electron microscope before and (d) after irradiation. (e) AFM image of the flake after irradiation. (f) Low-T confocal PL scan of the flake.

## Supplementary note 7: Continuous wave second order correlations

We have measured the second-order photon correlation of a SPE under continuous wave excitation at several powers around saturation (supplementary figure 7). The short-time correlation function exhibits a power-dependent bunching contribution decaying at timescales of order 100 ns. This observation is similar to what has been observed in SPEs from other physical systems [3, 4] and is consistent with the presence of a third metastable state of lifetime $\sim$ 400 ns [5]. The long-time $g^{(2)}(\tau)$ is constant up to timescales of milliseconds, confirming the absence of blinking dynamics in the range ns to ms. The stability at longer timescales is established by the intensity timetraces for $P < P_{sat}$ (see figure 2 of the main text), although at the highest powers, sizeable intensity fluctuations can be observed.

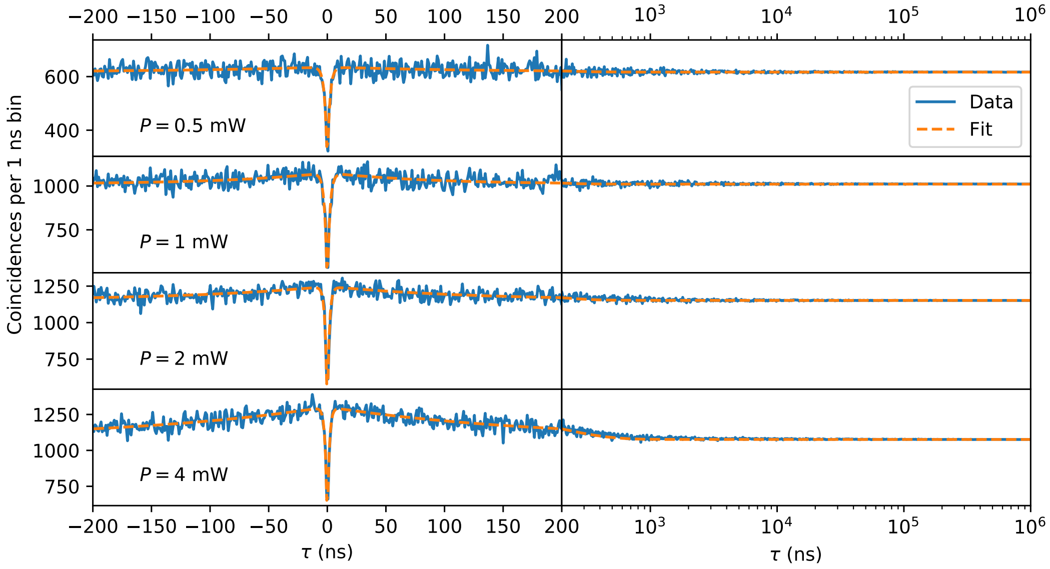


Supplementary figure 7: $g^{(2)}(\tau)$ measured at four different powers. Left panel: linear scale around 0. Right panel: logarithmic scale for the time axis. The orange line is a fit with a function of the form $g^{(2)}(\tau) = A - Be^{-|\tau|/t_1} + Ce^{-|\tau|/t_2}$

## Supplementary note 8: SPEs in 2D materials

In this section, we compare relevant properties of the main SPE families in 2D materials. For a fair comparison, count rates of SPEs coupled to optical microstructures have been discarded. Part of the table content has been extracted from reference [6].

### Supplementary table 1

| Material | Creation method | ZPL wavelength | ensemble linewidth | linewidth × lifetime | count rate (PL) | $g^{(2)}(0)$ | controlled positioning | room temperature operation | refs |
|---|---|---|---|---|---|---|---|---|---|
| hBN | annealing, e-beam | 550 – 780 nm | 300 meV | $\sim 1$ | $7 \times 10^6$ Hz | 0.05 | no | yes | [7, 8, 9] |
| hBN | strain | 530 – 625 nm | 175 meV | n/a | n/a | 0.27 | yes | yes | [10] |
| hBN | CVD growth | 568 – 590 nm | 75 meV | n/a | n/a | 0.2 | no | yes | [11] |
| **hBN** | **e-beam (this work)** | **436 nm** | **3 meV** | **TBD** ($< 300$) | $3 \times 10^5$ **Hz** | **0.1** | **yes** | **yes** | |
| $WSe_2$ | strain | 738 – 821 nm | 80 meV | $\sim 100$ | $1.6 \times 10^4$ Hz | 0.03 | yes | no | [13, 12] |
| $WS_2$ | strain | 610 – 681 nm | 100 meV | $2.6 \times 10^4$ | $1 \times 10^4$ Hz | 1 | yes | no | [13] |
| $MoSe_2$ | strain | 756 – 795 nm | 40 meV | n/a | $7 \times 10^3$ Hz | 1 | no | no | [14] |
| $MoS_2$ | He-ion beam | 696 – 721 nm | 31 meV | $6.6 \times 10^5$ | $4 \times 10^3$ Hz | 0.23 | yes | no | [15, 6] |

## Supplementary References


[1] Vuong, T. *et al.* Phonon-Photon Mapping in a Color Center in Hexagonal Boron Nitride. *Phys. Rev. Lett.* **117**, 097402 (2016) .

[2] Wigger, D. *et al.* Phonon-assisted emission and absorption of individual color centers in hexagonal boron nitride. *2D Mater.* **6**, 035006 (2019).

[3] Kitson, S.C., Jonsson, P., Rarity, J.G., Tapster, P.R. Intensity fluctuation spectroscopy of small numbers of dye molecules in a microcavity. *Phys. Rev. A* **58**, 620 (1998).

[4] Wang, J. *et al.* Bright room temperature single photon source at telecom range in cubic silicon carbide. *Nature Commun.* **9**, 4106 (2018).

[5] Martínez, L. J., Pelini, T., Waselowski, V., Maze, J. R., Gil, B., Cassabois, G. & Jacques, V. Efficient single photon emission from a high-purity hexagonal boron nitride crystal. *Phys. Rev. B* **94**, 121405(R) (2016).

[6] Klein, J. *et al.* Engineering the Luminescence and Generation of Individual Defect Emitters in Atomically Thin $MoS_2$. *ACS Photonics* **8**, 669-677 (2021).

[7] Tran, T. T., Bray, K., Ford, M. J., Toth, M. & Aharonovich, I. Quantum emission from hexagonal boron nitride monolayers. *Nat. Nanotechnol.* **11**, 37–41 (2016).

[8] Grosso, G. *et al.* Tunable and high-purity room temperature single-photon emission from atomic defects in hexagonal boron nitride. *Nature Commun.* **8**, 705 (2017).

[9] Dietrich, A. *et al.*, Observation of Fourier transform limited lines in hexagonal boron nitride. *Phys. Rev. B* **98**, 081414 (2018).

[10] Proscia, N. V. *et al.* Near-deterministic activation of room-temperature quantum emitters in hexagonal boron nitride. *Optica* **5**, 9, 1128-1134 (2018).

[11] Mendelson, N., Xu, Z.Q., Tran, T.T., Kianinia, M., Scott, J., Bradac, C., Aharonovich, I. & Toth, M. Engineering and Tuning of Quantum Emitters in Few-Layer Hexagonal Boron Nitride. *ACS Nano* **13**, 3132 (2019).

[12] Branny, A., Kumar, S., Proux, R. & Gerardot, B. Deterministic strain-induced arrays of quantum emitters in a two-dimensional semiconductor. *Nature Commun.* **8**, 15053 (2017).

[13] Palacios-Berraquero, C. *et al.* Large-scale quantum-emitter arrays in atomically thin semiconductors. *Nature Commun.* **8**, 15093 (2017).

[14] Branny, A. *et al.* Discrete Quantum Dot like Emitters in Monolayer $MoSe_2$: Spatial Mapping, Magneto-Optics, and Charge Tuning. *Appl. Phys. Lett.* 2016, **108** (14), 142101.

[15] Klein, J. *et al.* Site-selectively generated photon emitters in monolayer $MoS_2$ via local helium ion irradiation. *Nature Commun.* **10**, 2755 (2019).
